# Workplace Outcomes in Work-Disability Prevention Research: A Review with Recommendations for Future Research

**DOI:** 10.1007/s10926-016-9675-9

**Published:** 2016-10-27

**Authors:** Amanda E. Young, Eira Viikari-Juntura, Cécile R. L. Boot, Chetwyn Chan, David Gimeno Ruiz de Porras, Steven J. Linton, The Hopkinton Conference Working Group on Workplace Disability Prevention, Benjamin C. Amick, Johannes R. Anema, Elyssa Besen, Peter Blanck, Cécile R. L. Boot, Ute Bültmann, Chetwyn C. H. Chan, George L. Delclos, Kerstin Ekberg, Mark G. Ehrhart, Jean-Baptiste Fassier, Michael Feuerstein, David Gimeno, Vicki L. Kristman, Steven J. Linton, Chris J. Main, Fehmidah Munir, Michael K. Nicholas, Glenn Pransky, William S. Shaw, Michael J. Sullivan, Lois E. Tetrick, Torill H. Tveito, Eira Viikari-Juntura, Kelly Williams-Whitt, Amanda E. Young

**Affiliations:** 1 Liberty Mutual Research Institute for Safety, 71 Frankland Road, Hopkinton, MA 01748 USA; 2 Finnish Institute of Occupational Health, Helsinki, Finland; 3 EMGO Institute, VU University Medical Center, Amsterdam, The Netherlands; 4 The Hong Kong Polytechnic Institute, Hong Kong, China; 5 The University of Texas Health Science Center at Houston, Houston, TX USA; 6 Center for Health and Medical Psychology, Örebro University, Örebro, Sweden

**Keywords:** Disability outcome measures, Research priorities, Methods, Review

## Abstract

*Introduction* Outcome assessment is a central issue in work disability prevention research. The goal of this paper was to (1) ascertain the most salient workplace outcomes; (2) evaluate the congruence between business and science perspectives; (3) illustrate new perspectives on assessing longitudinal outcomes; and (4) provide recommendations for advancing outcome evaluation in this area of research. *Methods* The authors participated in a year-long collaboration that culminated in a sponsored 3-day conference, “Improving Research of Employer Practices to Prevent Disability”, held October 14–16, 2015, in Hopkinton, MA, USA. The collaboration included a topical review of the literature, group conference calls to identify key areas and challenges, drafting of initial documents, review of industry publications, and a conference presentation that included feedback from peer researchers and a question/answer session with a special panel of knowledge experts with direct employer experience. *Results* Numerous workplace work-disability prevention outcome measures were identified. Analysis indicated that their applicability varied depending on the type of work disability the worker was experiencing. For those who were working, but with health-related work limitations (Type 1), predominant outcomes were measures of productivity, presenteeism, and work-related limitations. For those who were off work due to a health condition (Type 2), predominant outcomes were measures of time off work, supervisor/employee interactions, and return-to-work (RTW) preparation. For those who had returned to work (Type 3), predominant outcomes were measures of presenteeism, time until RTW, percentage of work resumption, employment characteristics, stigma, work engagement, co-worker interactions, and sustained or durable RTW. For those who had withdrawn from the labor force (Type 4), predominant outcomes were cost and vocational status. *Discussion* Currently available measures provide a good basis to use more consistent outcomes in disability prevention in the future. The research area would also benefit from more involvement of employers as stakeholders, and multilevel conceptualizations of disability outcomes.

## Introduction

Evaluating the outcome of any preventive intervention program is integral for program development and the future choice of initiatives. Work disability is costly for workplaces, families, and society at large with enormous expenditures every year [1, 2]. Workplaces invest sizeable amounts of resources to implement preventive interventions. Careful assessment is of central importance in the evaluation and comparison of interventions. To achieve a sound evaluation, relevant outcomes need to be identified and measured.

In this paper we address the question of how outcomes might best be assessed from a scientific as well as from a business perspective. As reviewed in earlier works [3], considerable effort has been made by the scientific community to develop instruments to measure work-disability and return-to-work outcomes; however, these tend to reflect the interests of scientists. The extent to which they resonate with employer groups is largely undocumented. Within the scientific community, there is emphasis on psychometrically vigorous instruments that assess outcomes such as symptoms and functions that are measured over periods of time. However, the workplace may have a different perspective. They may be interested in immediate results like the cost of the program and how much it disrupts production. It is our contention that in order to advance workplace disability prevention research, it is important to better understand the business community’s perspectives as well as the scientific.

Within both the business and scientific communities, outcomes are usually defined in relation to goals. Consequently, we define outcomes as the degree to which the goals of the work disability prevention (WDP) program are achieved. Because programs may have the goal of tackling certain risk factors (e.g., work limitations, workplace relationships and work engagements) measures of these are relevant as outcomes, with this being especially true for those that may be amenable to change. A further consideration is that WDP goals often focus on health and the ability to work productively. In many cases, both subjective and objective measures are available, and sometimes necessary, to evaluate goal attainment. Establishing clear goals is an important program feature as this enables the use of appropriate outcome measures. For the purpose of this paper, WDP outcomes measures should be understood as measures reflecting the effects of, formal or informal, work-disability policies and procedures addressing physical, social and/or psychological aspects of the workplace.

Given that is it is likely that science and business have different goals in evaluating outcomes, it is important to review measurement from both perspectives. To advance the understanding of currently available workplace WDP measures, identify disparities between employers and scientists and to pave the way for future research, this paper was written with the intention to:

- Ascertain the most salient workplace WDP outcome measures and evaluate their strengths and weaknesses
- Compare the congruence of employer and science perspectives
- Provide recommendations for advancing outcome evaluation in workplace-based WDP research

## Method

With a goal toward improving future research of employer disability prevention strategies, the authors participated in an invited 3-day conference, “Improving Research of Employer Practices to Prevent Disability”, held October 14–16, 2015, in Hopkinton, MA, USA. Methods and general proceedings of the conference are described in the introductory article to this special issue [4]. The authors of the present article represented a sub-group tasked with examining w*orkplace outcomes in work*-*disability prevention research.* We were asked to address the question: “What are the principal workplace outcome measures in disability prevention research?” The overall purpose and design of the work disability symposium is described in the Introduction to this Special Issue [4].

We recognize that there are many stakeholders involved in work-disability prevention and that an integrated multidisciplinary partnership between the diverse groups (e.g., employers, workers, clinicians) is an effective approach to developing successful and efficient WDP strategies [5]. However, we were charged with focusing on measures addressing workplace features, including organizational policies and procedures, impacting and impacted by workers’ work-disability with a specific focus on the employers’ perspective. As such, WDP outcomes that are not explicitly workplace-related have not been included for discussion in this current work.

The understanding that WDP initiatives vary depending on where the individual is in the work-disability spectrum [6] provided a conceptual framework for our analysis. Based on the developmental conceptualization of return to work [6], we categorized work disability into four different types.

1. *Working, but experiencing health*-*related work limitations*—the affected person is still working, but is experiencing symptoms that are interfering with his/her work.
2. *Off work due to health condition*—the affected individual is absent from work due to a health condition.
3. *Returned to work with work limitations*—the affected individual is back at work, but experiencing work restrictions.
4. *Withdrawn from the labor force*—the affected individual is withdrawn from employment due to his or her health condition.

**Fig. 1:**
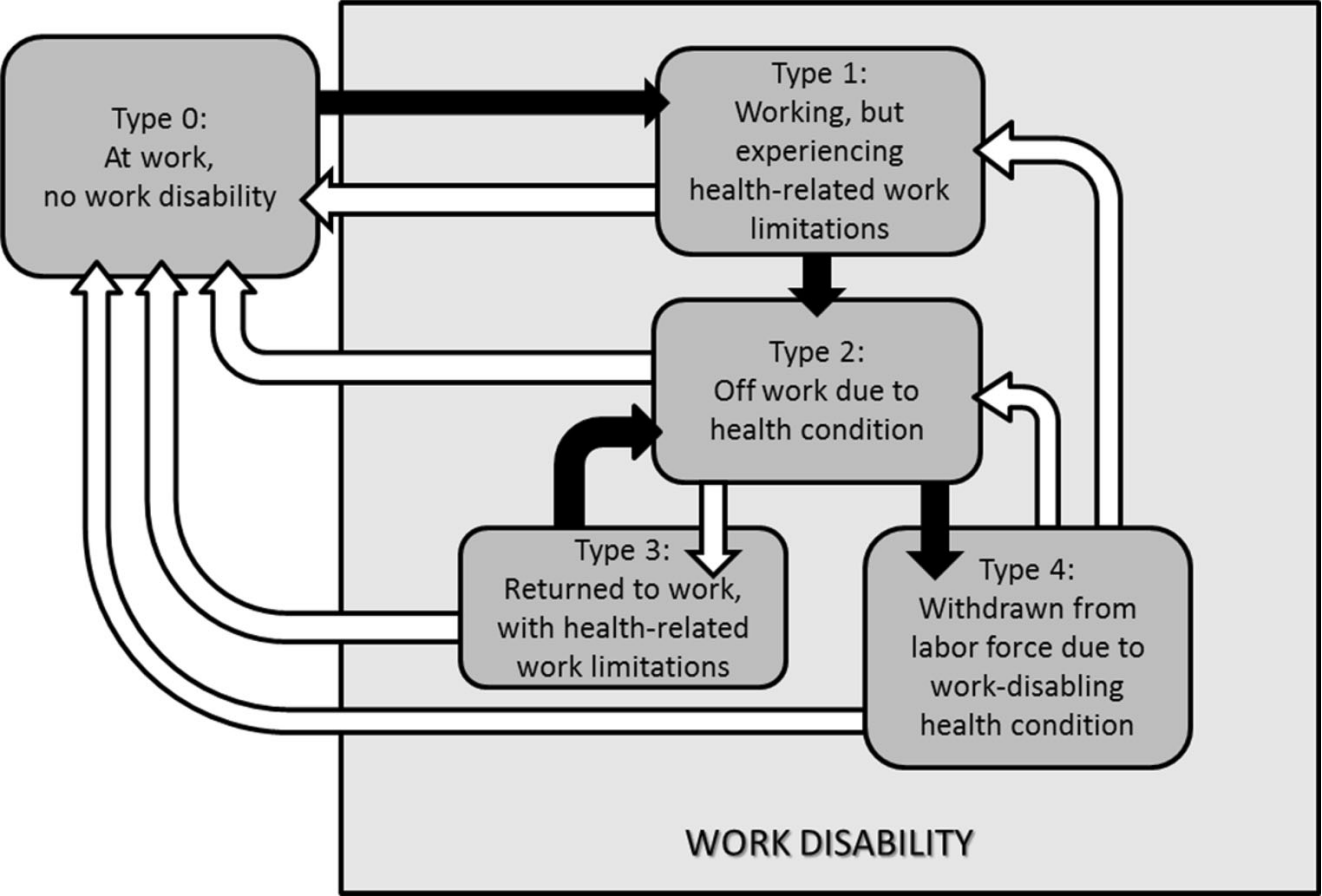
Diagrammatical representation of the various types of work disability, based on earlier works describing the developmental nature of return to work (Young et al. [6]). As illustrated by the *arrows*, the categorization recognizes that people can move between WD types. In terms of work disability prevention, the *black arrows* indicate negative change, and the *white arrows* indicate positive change

It should be noted that in suggesting this conceptualization, we feel it is important not to categorize the different work disability (WD) types as “phases” This is because we do not want to imply that people progress through each WD type. However, the categorization recognizes that people can move between WD types, and that interventions can help persons shift from one disability type to another. An illustration of this is included in Fig. 1 in which the arrows indicate movement: black = negative in terms of WD, white = positive in terms of work disability.

Prior to the conference, we conducted a narrative review of the scientific and gray (non-scientific business) literature reporting outcomes we felt relevant to workplace disability prevention research. The review was led by our chosen conceptual framework and professional experience in the domain. Our group was formed by the conference organizers based on our interest and experience in the field. We were instructed to address a particular topic area, and conduct a descriptive review, rather a comprehensive assessment of the literature. As such, the papers cited should be viewed as illustrative examples, and not an exhaustive representation of all works measuring workplace WDP outcomes. It should also be noted that when reporting our findings, unless it is specifically stated otherwise, citations refer to works from the scientific literature. Throughout the paper we comment regarding employers’ interest in workplace outcome measures that have been used in the scientific literature; however, it should be noted that not all workplace measures are likely to be of (equal) interest to this stakeholder group.

Once we had decided the outcomes on which we wanted to focus, and obtained input from conference participants, our group tasked itself with summarizing the various outcome groupings and making recommendations for future research. To help researchers in the field to better understand the potential strengths and weaknesses of the different WD measures, we conducted an analysis of the WD outcome groupings. To facilitate researchers and other stakeholders, such as employers, in their decision making regarding which RTW outcomes to employ in their studies, we assessed each of the groupings using six criteria. These were: (1) psychometric properties—the extent to which the measures for RTW outcomes have evidence of validity and reliability; (2) context independence—the meaning and interpretation of RTW outcomes is consistent across different systems and settings; (3) potential for trajectory/RTW process research—the capacity of the measures to be utilized for assessing changes in disability and RTW outcomes over time; (4) availability—the extent to which the data involved in the measures can be easily available; (5) cost—the amount of cost involved in capturing the data required in the measures; (6) employer interest—our subjective rating of the extent of employer interest in the RTW outcome.

Four of the authors of current paper reviewed the strengths and weaknesses of the WD measures groupings as part of the Hopkinton Conference program [4]. Groupings were rated according to a five-point rating scale: “+++” (yes—high), “++” (yes—medium), “+” (yes—low), “No” (no) and “NA” (not applicable). Each of the four group members presented their ratings verbally. If there was disagreement between ratings, this was discussed until consensus was achieved. The remaining authors reviewed the assigned ratings, and were in agreement with the assessments made. Unfortunately, it was beyond the scope of the current exercise to go into detail regarding how best to measure each of the identified constructs.

## Results

The results of our review revealed measures that included those that allow for the assessment of whether or not an intervention was successful in terms of helping a person stay at work, decreasing the amount of work absence, and returning workers to productivity. These are described below. Table 1 contains a summary of workplace outcome measures by WD type that have been referenced in the WDP literature, together with the results of our strength/weakness analysis.

**Table 1:** Work-disability prevention outcomes by work-disability type

Work-disability prevention outcomes	Paper citing outcome	Assessed for psychometric properties	Context independent	Potential trajectory/RTW process outcome^a^	Availability	Cost	Employer interest
WD Type 1: before sickness absence							
Productivity	[8–10, 13]	+++	+++	+++	+++	+	+++
Presenteeism	[12]	+++	+++	+++	+	+++	+
Work limitations and abilities	[16–31, 88]	+++	++	+++	+	+++	++
WD Type 2: off work							
Time off work	[33–35]	·	–	+++	+++	+	+++
Employee-employer interactions	[37]	+++	++	+++	+	+++	++
RTW preparations	[48]	+	+	+	+	+++	++
Work absence recurrence	[33, 83, 84]	·	–	−/+	+++	+	+++
WD Type 3: back at work							
Time: until RTW, back at work, until sustained RTW	[51, 52, 99]	·	–	−/+	+++	+	+++
Duties, position and employer	[53–57]	·	–	+	+	+++	++
Co-worker interactions	[64]	+	++	++	+	+++	+
Work engagement	[60]	+++	+++	++	+	+++	++
Stigma	[59]	+++	–	+	+	+++	+
Sustained RTW	[67–70]	·	–	−/+	++	++	++
Durable RTW	[77]	+	–	−/+	+	+++	+
WD Type 4: withdrawn from labor force							
Labor force participation	[78–80]	–	–	+	+++	+	–
Vocational status	[79]	–	–	+	+++	++	–

*RTW* return to work

*Legend* “+++” = High; “++” = Medium, “+” = Low; “−” = No; “·” = Not Applicable

^a^Outcomes marked “−/+” indicate those that we assessed as not suited for WD trajectory research, but have the potential to be used as RTW process outcomes. *Note* Outcomes that are described earlier in the table are also applicable to later types of work disability (e.g., productivity, presenteeism, work limitations and ability, employee–employer interactions), but for ease of presentation, are not duplicated

### Type 1—Working, But Experiencing Health-Related Work Limitations

WDP initiatives for people with this type of WD focus on preventing needless work disability by helping people experiencing symptoms stay employed. Health-related work limitations are defined as limitations to the worker’s ability to do their job imposed by his or her health condition. Within the current context, the term “limitations” is used to encompass both activity limitations and participation restrictions as conceptualized in the International Classification of Functioning (ICF) [7]. That is, it refers to both difficulties performing a particular task or action (activity limitations), as well as difficulties participating in work (participation restrictions). Workplace WDP outcome measures relevant within this WD type include:

#### Productivity

Perhaps one of the most important indicators is worker productivity. Instruments that have been used to assess productivity include assessments based on recorded productivity data [8] and measures based on individuals’ assessment of how they performed their duties [9, 10]. Of particular interest is the Occupational Role Questionnaire which consists of two scales—productivity scale and satisfaction with work scale [10].

#### Presenteeism

A recent development within the RTW literature is a focus on presenteeism, which is the act of attendance at work while sick, also referred to as at-work productivity loss due to health problems [11]. In contrast to productivity focusing on what a person at work can do, presenteeism focuses on what a person at work cannot do. Presenteeism can lead to productivity losses which can be easily overlooked. Recent studies have shown that productivity losses at work due to presenteeism are high and actions are needed to reduce these losses [12]. As presenteeism focuses on at work productivity loss, measurement instruments focusing on productivity might actually focus on presenteeism. For example, the Worker Limitations Questionnaire incorporates limitations with handling time, physical limitations, mental limitations, and limitations regarding handling work demands [13]. Additional self-report instruments that have been designed over the past few years to measure the impact of illness on productivity at work and/or in non-work activities include the Endicott Work Productivity Scale, Health and Labor Questionnaire, Health and Work Questionnaire, and the Health and Work Performance Questionnaire [14]. A more comprehensive review of presenteeism measures is contained within the chapter by Amick III and Gimeno [15].

#### Work-Related Limitations and Abilities

Health impairment often leads to work ability impairment. To understand the extent of the problem, there is a need to gain an understanding of the health-related limitations of symptomatic employees. In addition, for people who are working through a period of WD, it is necessary to determine what that person can and cannot do. Measures are available to assist with determining this. Within the RTW literature, a plethora of instruments have been used to assess various aspects of work ability: physical [16–20], mental [16, 18, 21, 22], and, functional [16, 18, 23–25]. Among these instruments, a distinction can also be made between those operating outside of a specific job context [16–25] and those that include specific job requirements [23, 26–32]. Instruments incorporating specific job characteristics are known as functional or work capacity assessments. While we mention outcome measures of this type in this section on WD Type 1, it should be noted that assessment of limitations and abilities are applicable to all types of WD.

### Type 2—Off Work Due to Health Condition

WDP initiatives for WD of this type are focused on returning the worker to the workplace. Outcomes that have been used include:

#### Time Off Work

A commonly used measure in workers’ compensation research is time off work. Examples of studies using this measure are found in the literature [33–35]. Although useful for defining WD in terms of acute, sub-acute and chronic [36], this measure provides limited understanding of the reason why the person is off work. As described below, additional measures can be used to gather this detail.

#### Supervisor/Employee Interactions

Supervisors play a key role at the workplace since they have immediate contact with the employee [37]. Since many RTW programs include workplace factors (e.g., work demands), supervisors are an important link that can influence program success [38, 39]. One particular example is the case of modified duties, which has been found to be an effective method for improving RTW outcomes [39]. Supervisors are typically involved in providing various forms of modified duty [39]. The interaction between the supervisor and employee is also thought to be vital [40]. However, supervisors and others involved in facilitating RTW may have very different backgrounds and often have received only minimal training [41, 42]. Not only are communication strategies of interest, but also relevant is how the supervisor and employee interact to solve problems to facilitate a modified return to work [43]. While skills for supervisor–employee communication and problem solving may be central for successful RTW, their measurements are yet to be standardized.

#### RTW Preparations

RTW following a period of work disability health condition has been described as an interplay between bio-psychosocial factors surrounding the workers and employers [44]. Previous studies have indicated that worker perceptions regarding their functional capacity [45], psychological readiness for RTW [46] and RTW expectations [47] are the significant predictors of successful RTW. As such, these can be used as indicators of RTW preparation and as outcomes in intervention research aiming to move workers closer to the goal of returning to work. Employers’ receptivity, timeliness of RTW arrangements, and the availability of accommodation needed to promote a safe working environment for the injured workers [48] are indicators that can be used to assess whether RTW preparations are adequate. This may also involve how employees view the success of the rehabilitation for a workmate (e.g., how well the return is orchestrated). Because the readiness of the workplace is a key factor for successful RTW, the measurement of RTW preparation involves all employees, even those without a health condition. Examples of instruments include the Lam’s Assessment of Employment Readiness (LASER) [49] and the Readiness for Return-To-Work (RRTW) scale [50], both of which tap into the sick-listed workers psychological readiness for RTW. These and other outcome measures of this type are often used by clinicians when designing return-to-work interventions.

#### Work Absence Recurrence

Work-disability recurrence has been the topic of much investigation. Results indicate that recurrences contribute disproportionately to the total burden of work-related work-disabling conditions. As an example, in the case of nonspecific low back pain, recurrence of the condition adds to the cost of injury through both additional care seeking and work disability. Findings imply that those who have recurrences may be an especially important target for secondary prevention efforts [33].

### Type 3—Back at Work

The outcomes used mirror many of those mentioned in our section on measures that are relevant prior to work absence (i.e., WD Type 1) and include outcomes such as work-related limitations, abilities and productivity. However, additional considerations include:

#### Presenteeism

Measures of presenteeism are also relevant for WD of this type. Depending on a worker’s circumstances, presenteeism might be expected if a RTW is implemented with the intention of shortening work absence and facilitating reincorporation of worker into his or her job. Alternatively, presenteeism would probably not be expected when the worker is fully recovered from the condition that caused time away from her or his job in the first place. Although the intention behind applying the measure might vary depending of the worker’s circumstances, the measures used for assessing presenteeism for persons with WD Type 1 (see above) are also applicable for people who are back at work (i.e., with WD Type 3).

#### Time Until Return to Work

Past RTW research has included instruments evaluating time to return to workplace and time to maintain RTW [51, 52]. Differentiations are made between simply returning to work, returning part-time and returning in a fully-functioning capacity.

#### Proportion of Time at Work

This outcome can be used as an overall measure to describe the amount of work, i.e., proportion of full-time work during a time period. This measure can be meaningful in some jurisdictions where work ability is certification assessed as a proportion of time. If proportion of work time is used as an overall measure of periods of work participation and sickness absence, a drawback is that it does not contain anything about the timing and length of the periods. In such a situation, this measure should be accompanied with other outcomes, e.g., trajectory analysis (described later in this paper).

#### Employment Characteristics

Return-to-work outcomes can also be described in terms of the type of actions undertaken by workers resuming employment. Depending on research aims, the focus can be placed on details such as the type of duties performed (full, light, or modified, i.e., with accommodations) [53, 54]. Distinctions can also be made between returning to the same or a new job [55, 56] and the same or new employer [57]. These outcomes can be of particular interest in an applied setting as there is often a hierarchy of preference such that a return to the pre-absence employer, in the same job, at the same capacity is seen as the best RTW scenario [58].

#### Stigma

Perceptions of stigma following RTW have also received research attention. In a paper that discusses injured workers’ points of view, workers reported a range of impediments experienced in the return-to-work process that created considerable stress and concern. This included stigma associated with a registered workers’ compensation claim, disrespectful communication from service providers, and a suspicious response to their health condition by the employer, co-workers and some professional service providers  [59].

#### Work Engagement

Research has also looked into levels of work engagement. Examples of studies include: an exploration of work engagement in employed tumor-free cancer survivors compared to matched controls from the general population which found no difference [60], and a study of traumatic brain injury patients 1–2 years after discharge which found that their level of engagement was related to acceptance of disability [61].

#### Co-worker Interactions

The work reintegration process can set several requirements for co-workers’ support for the returning worker such as taking over tasks that the returning worker is unable to do and, sometimes even, partly organizing or managing the reintegration. The co-workers’ capacity to provide support varies by the quality of the work culture, i.e., how supportive the culture is and how collectively the work to be performed is perceived [62]. Other important factors are the perception of the fairness of the accommodations for the returning worker as well as the duration of the arrangements [63]. For a short period of time, undesirable workloads can be accepted; however, if the situation continues for weeks, it may no more be tolerable [62]. The effects of work reintegration on a co-worker can be positive, such as learning new skills and getting a sense of achievement. However, detrimental effects have often been reported, such as an increase of stress, contracting illness or even leaving the workplace [62]. Overall, studies emphasize the importance of social relations, especially with co-workers, in the success of the return-to-work process [64]. While these qualitative studies identify the importance of worker-worker interactions, the extent to which measures have been designed to assess these outcomes is limited. An instrument to measure workplace social support for workers with disability, consisting of 11 items on co-worker support, has been developed [65]. There are also instances where a single-item measure has been used to assess co-worker support [e.g. 66].

#### Sustained RTW

Sustained return to work for at least 28 days has been used in the majority of recent randomized control trials, e.g., from The Netherlands [67], Denmark [68], Norway [69] and Finland [70]. The basis noted in the Dutch studies is that 4 weeks is a natural time period of interest, since a recurrence within that period is included in the initial sickness period in the Dutch Sickness Benefits legislation. A corresponding rule exists in the Finnish Health Insurance Act, according to which a recurrence within 30 days with the same diagnosis as the previous will give right to continued compensation by the Social Insurance Institution. While 28 days is the most commonly used timeframe, studies have used other criteria including 6 months [71] and 2 years [72]. Another measure that taps into the concept of sustained RTW is the measure labelled “return to work in good health” [73], which is based on a combination of patients’ occupational status, functional limitations and recurrences of work absence over a given timeframe (1–2 years). Researchers have also used a measure that includes an assessment of whether or not employment participation was maintained or improved in comparison to an earlier point in time [74].

#### Durable RTW

It has been suggested that when measuring return-to-work success, commenting on the potential for longer term success is also of importance [75]. While this suggestion is generally accepted, there has been limited research to measure this construct. A measure labelled “durable RTW,” which is the proportion of injured workers who had returned to work and were still working at the time of interview, has also been employed [76]. Research on factors to consider when attempting to determine if a RTW is durable indicate the importance of perceived risk of physical and/or psychological harm, the ability to perform the work, the demand within the context of the environment and the extent to which the RTW is consistent with personal needs and circumstances [75]. Along the same lines, results of a prospective study of people returning to work after undertaking vocational rehabilitation indicated that those who were worried that symptoms might interfere with their ability to continue in the job, who had difficulties with the job’s physical demands and a strong desire to leave their current job were less likely to be employed or in the same job at the time of follow-up [77].

### Type 4—Withdrawn from the Labor Force

The outcomes of relevance to this WD type look at withdrawal from any workplace and movement out of the labor force (i.e. not working and not looking for work). Studies using these types of outcome have studied the contribution of diseases, such as arthritis, to non-participation in the labor force [78], return to work following spinal cord injury [79] and labor-force participation in Canadian adults with activity limitations [80]. Generally, people are defined as either in the labor force (employed or unemployed and looking for work) or not. For those not in the labor force, outcome sub-categories would include: unemployed and not looking for work, movement [36] to some type of social security benefit, or self-funded retirement, attending an educational institution, home duties and caring for children. For employers, this outcome is likely to be of interest as it relates to the likelihood of the worker returning to their establishment. Those who state withdrawal from labor force participation are probably unlikely to return to their pre-WD job, indicating to an employer that there is a need for staffing review. Other similar measures include vocational status [79], and vocational mode [81].

### Overarching Measures

An important outcome for employers relates to the costs of programs and how these are sufficiently offset by reductions in disability and health care costs or concomitant improvements in worker productivity. To build a business case, researchers have included economic evaluations alongside controlled or pragmatic trials of new or experimental WDP programs. The purpose of these economic evaluations is to identify, measure, and compare costs and health consequences of two or more programs or interventions (including comparison with nominal or usual practices). In most countries, employers would bear the financial consequences of lost worker productivity and the administrative burden of rehiring and training, but other costs associated with disability and health care expense may or may not be relevant to the employer depending on national differences in health insurance and disability systems. Following is a brief summary of the economic evaluations that are, in our opinion, likely to be of greatest importance to employer groups. We note that additional economic evaluation approaches exist, and the relevance of the approach will vary depending on stakeholder priorities and contextual backgrounds; however, addressing this in detail was beyond the scope of the current exercise.

Economic outcomes can be distinguished into four major types: *costs, cost*-*effectiveness ratio (CER), cost*-*utility ratio (CUR) and return on investment.* An overview of the net costs associated with a program or intervention requires a systematic collection of all costs associated with that program or intervention. The CER is useful to compare the costs of an intervention or program with its effects as expressed by a common health effect. The CER is calculated by the difference in costs between the intervention and a control intervention, divided by the difference in effects between the two interventions. This ratio can be expressed as the dollar value per day a worker returns to work sooner. The CUR enables us to compare different interventions and/or different groups. Therefore, the effect of an intervention needs to be expressed in utilities such as e.g., Quality Adjusted Life Years (QALYs).

Overall, the costs associated with the programs in relation to production gain benefits (days lost from work, work productivity) that they generate are needed to calculate these outcomes. This information is potentially available from company records, but it is important that the presence is confirmed from the very start of the intervention as it is sometimes needed to perform additional actions (e.g., questionnaires) to retrieve the information. Moreover, these administrative data offer many opportunities to study trajectories in costs and benefits.

### Stakeholders Input

During the conference, we discussed the above-mentioned outcomes with an audience consisting of scientists and a special panel of employers, policy makers, and practitioners. In general, relevant outcomes were shared by all stakeholders. A summary of our subjective rating of level of stakeholder interest in the various outcomes identified in the scientific literature are contained within Table 1. In addition to the outcome measures we identified, the stakeholder panel also mentioned the importance of performance of suppliers of WDP programs (vendors) and compliance with internal organizational processes. Examples of additional measures included disruption to production, employee satisfaction, safety and staff turnover. These outcomes often develop over time, and may need more time to become visible than which is generally available for employer-based effect evaluation studies. Therefore, it is likely that the feasibility of including these in WDP studies is low. However, scientists should be aware of these effects and should explore ways to include these outcomes in research as this will reduce the gap between science and practice. An interesting divergence occurred in relation to the concept of presenteeism which the employer panel did not rate of high interest. When asked to elaborate, the indication was that this was not really viewed as a cost of work-disability, with more pressing matters, such as productivity and compliance, being of greater interest.

## Discussion

Our review revealed workplace WDP outcomes that were many and varied, and we found both consistency and divergence as it related to scientist and employer interest. With that said, it should be noted that what we have presented is an overview and it is likely that the level of interest is not consistent across contexts. For example, outcomes of interest to employers may vary depending on factors such as condition etiology (occupational vs. non-occupational), who pays (employer, worker, or society), corporation size (large vs. small) and the worker’s skills (highly specialized vs. low skilled). In addition, interest is likely to be influenced by the role the employer representative plays within his or her organization. For example, human resources management may be more likely to be interested in policy compliance, whereas line managers are likely to be more focused on productivity and morale. When conducting WDP research, it is important to recognize these differences and incorporate them into study designs.

Although an attempt was made to address which measures have been assessed for reliability and validity, it was beyond the scope of this review to comprehensively assess levels of measure development. While outcomes are referenced in the literature, the degree to which measures have been developed varies greatly. It is our opinion that WDP research would greatly benefit from a dedicated effort aimed at developing a set of field-specific measures that can be applied depending on the aims of research, but would allow for cross context comparisons.

With regards to contrasting business and scientific approaches, we believe that there is a need to bring the views of employers and scientists together in order to achieve better outcome assessment. Based on our review, employers have had a primary interest in outcomes like the direct costs of the program, the extent to which the intervention will disrupt production, and the immediate benefits of the program for the workplace. On the other hand, scientists are likely concerned with the integrity of the intervention, the underlying mechanism involved, the process over longer periods of time, and the effects on the health of workers. Luckily, we observed some overlap in interest areas (see Table 1). This shared interest should provide fertile ground for workplace collaboration and engagement.

Our analysis of workplace WDP outcomes that appear in the literature indicates that WD Type 3 has the largest number and variety of outcomes; however, these outcomes are not necessarily of the greatest interest to employers, who appear more focused on WD Type 2 outcomes. Measures of WD Type 4 are few and do not appear to be of great interest to employers. This is, perhaps, not surprising as by this point, employers have had to deal with staffing and productivity issues, with WD costs shifting to other payers (e.g., welfare and social security systems). If we really want to focus on WD prevention, more effort needs to be put into measuring what is going on when people have WD Type 1. Because employers are likely less interested in this, we should focus on measures that are readily available within company registrations and work on monitoring and surveillance systems to observe trajectories. This would be helpful to detect unfavorable changes in Type 1 outcomes at an early stage.

While our presentation of workplace outcomes is organized by WD type, some outcomes can be used for one or more types of work disability. And sometimes it can be of interest to follow the development of an outcome over time at repeated time points. As has been indicated in Table 1, a few outcomes are appropriate for this and can, accordingly, be used as a basis for a trajectory. Trajectories can incorporate dynamic patterns for quantifiable elements over time. They can also identify distinct latent groups of subjects who tend to have a similar profile [82]. For example, work participation can be followed over time as work participation status (at work/off work) or proportion of time at work. Moreover, ordinal scales can be created to incorporate different grades of work participation (e.g., “not working,” “part-time working,” “full-time working”). Trajectory analysis can be done with rather small data samples. Similarly, RTW patterns have been examined in investigations focusing on recurrence of work disability [33, 83, 84] and patterns of employment following a work-related health condition [85, 86].

There are also measures that can be used for testing movement in the RTW process. As defined and elaborated upon in earlier works [6, 77], the RTW process can be described as being dynamic and bi-directional. It is said to be dynamic because the worker moves through different types of WD from when they begin experiencing health-related limitations until they achieve their final RTW status (see Fig. 1). Stages of RTW can be categorized as “off work due to health condition” (WD Type 2), “returned to work, with work limitations” (WD Type 3), and “at work, no work disability” (WD Type 0). Unsuccessful RTW process would result in workers “withdrawn from labor force due to health condition” (WD Type 4). When at work, workers can be with “no work disability” (WD Type 0) or “experiencing health-related work limitations” (WD Type 1). It is bi-directional because workers can, due to changes in the intrinsic factors such as physical or emotional health or extrinsic factors such as job or workplace demands, progress (e.g., from WD Types 3 to 0) or regress (e.g., from WD Type 3 to 4) through the RTW process. Difficulties with progressing in the RTW process may deter the workers from engagement in the workplace and, in the worst scenario, result in them being unable to move to a place where they are not experiencing work disability, hence withdrawn from the labor force. Within this context, WD outcomes are useful for expressing and predicting the movements of workers across the various stages of RTW. When testing RTW progress in terms of moving from being off work (WD Type 2) to returning to work with limitations (Type 3), it is useful to include an assessment of the time it takes for this occur (identified in the current review as “time off work” [33–35]). In addition, one could also measure “RTW preparations” [48] and “supervisor/employee interactions” [37]. The measures are useful for describing and predicting RTW movements from return to work with limitations (Type 3) to at work with no disability (Type 0), including “proportion of time at work” [87] and “presenteeism” [12]. The workers’ movements between no disability (WD Type 0), working while experiencing health-related limitations (WD Type 1) can be assessed in terms of “productivity” [8–10, 13], “presenteeism” [12], “work-related limitations and abilities” [16–31, 88] and readiness of workers for increasing work hours or work duty (e.g., modified C-LASER [49]).

A critical step in conducting successful workplace WDP research is employer engagement. The role of the above-mentioned economic outcomes on the decision of employers to engage in programs directed at the prevention of work disability should not be overestimated. First, the entity making the investment is often different from the entity receiving the (positive) return. In the case of the trial by Lambeek et al., the health insurer needed to invest in a return-to-work program, whereas the employer received the benefits resulting from earlier return to work [89]. This is a major barrier for WDP implementation. The question who pays differs between countries. In the US for example, the employee will not receive salary in case of non-work-related sick leave, whereas in e.g., the Netherlands, the employer is responsible for paying the salary of the employee on sick leave for the first 2 years. This has major consequences for who will benefit from investment in a return-to-work program. Second, many (positive) aspects of work disability prevention programs cannot be caught in measures that can be included in economic evaluations. It is questionable whether return on investment should always be what is most important. Sometimes programs that do not make money, or even require an (acceptable) investment, are worth implementing, as they may contribute to positive processes that are difficult to include in economic evaluations. For example, employers may become more attractive for talented workers, or have a more positive image to society that may enhance profits. In addition, improved well-being likely has ripple effects in terms of improving productivity and decreasing presenteeism [90, 91].

The issue of cost versus benefit also applies to outcome measure usage. We note that outcomes that provide greater insight and that are, perhaps, more interesting to scientists, are often more difficult and costly to collect. We also note that subjective outcome data tends to be less available and more expensive to collect. In WDP research, subjective measures are commonly used to evaluate a wide range of variables including presenteeism, productivity, and quality of workplace accommodations. On the other hand, “objective” data is drawn from official records, e.g., from an insurance agency (number of compensated days), number of visits for health care covered by the workplace, or production output. While objective data is often thought of as having higher validity than self-reports, which are subject to bias and recall error, there can be problems with either source. Because of the errors in the administrative systems and the difficulties in putting the files together, self-reports may be just as accurate as the administrative data [92–94]. Another problem with administrative data is that it usually does not cover an important aspect of evaluation, namely how the worker experiences the intervention and its consequences on health. As such, subjective data adds richness to understanding that cannot be achieved with objective data alone. Thus, depending on the research aims, there will be times when increased costs will need to be born to fully illuminate the impact of the study variables on WD outcome.

An additional consideration as it relates to evaluation of WDP initiatives is that a control group is not always readily available. As such, effect size can be difficult to determine. Demonstrating meaningful change is important for employer engagement. WDP researchers need to be creative in terms of evaluating their results. This could be various forms of benchmarking or other methodologies that allow for an interpretation of the effects of the program. An example comes from the RE-AIM framework, which advocates for the inclusion of measures of (1) the extent to which the intervention reaches the target population, (2) efficacy, settings, or institutions, (3), adoption by target staff, (4) implementation consistency, and (5) maintenance of intervention effects [95]. Not only would including such measures give stakeholders additional information upon which to judge the value of a workplace WDP initiative, it would also provide researchers with information regarding the likelihood of intervention success beyond the research setting.

Finally, we note that another key to program success is the timely reporting of results that are of interest to stakeholder groups [96]. Although researcher and employer interests will likely stress different aspects, it is our opinion that workplace WDP research would benefit from integration to achieve interim outcome evaluation and reporting opportunities. Ideally, this would involve multidimensional outcome assessment of a range of variables over extended periods of time. Doing this would benefit both researcher and employer groups. For scientists, this would facilitate an understanding of the immediate impact at work and the underlying organizational factors affecting implementation. For employers, it could provide a valuable understanding of the central factors involved and how they might be changed to benefit the worker and the workplace. In particular, since preventive interventions have a suspected impact over long periods of time, the true benefit of a program might need to be assessed accordingly.

### Directions for Future Research

Although there are a host of workplace WDP outcome methods and observational techniques, our review and analysis suggest that here are several lines of investigation that would significantly improve WDP research. While researchers and employers have attempted to measure outcomes from different perspectives, much could be gained by integrating outcome evaluation. Based on our review and professional experience, and specific to workplace WDP research, we recommend the following:

*Use multilevel sampling* that would include the perspectives of various people in an organization. Measures are needed that would tap into relevant experiences of people at different levels. This might, for example, help to assess any disruptions in production, side-effects, or benefits, e.g., a better work environment.

*Further work into measure development*, especially as it relates to more complex and subjective outcomes, would facilitate a better understanding of the work disability experience. In particular, we note that employee-employer interaction is very important in the RTW process [97], and that measures of this are underdeveloped. Similarly, worker-coworker interactions can play an influential role in terms of supporting/delaying RTW [40, 98], but measures to capture this are largely absent. Health economic measures also need to be developed further to meet the needs of both scientists and employers.

*Consensus on a composite set of outcome measures*. In our opinion, it would benefit the field if researchers and practitioners were to agree on a core set of outcome measures that would be applicable for various groups of workers and various work environments and would allow a comparison of findings within and, possibly, between jurisdictions. Such a core set should include outcomes that can relatively easily be translated into monetary terms, for instance presenteeism, productivity and proportion of time at work.

*Use of coordinated measures that are relevant to both researchers and employers*. While many of the outcomes used in the scientific literature were identified as of interest to employers, we noted outcomes pertinent to employers that do not appear in the scientific literature (e.g. disruption to production, employee satisfaction, safety and staff turnover). We also noted that measures used in the scientific literature were not always on the top of the agenda within the business arena. Care should be taken to include measures that are important to both groups.

*Employ measures that are applicable from the initiation of the program through long*-*term follow ups*. Employers may want to monitor progress from the start, but many current measures are of value primarily after the program has been in use a longer period. Similarly, an important outcome is how the program works on a long-term basis. Thus, measures are needed that are not only relevant over time, but that can be repeated continually over time. While this paper presents some outcomes of this type, more measures are needed to capture how the results develop over time.

*Evaluation and effect interpretation*. Evaluation is not complete when the data is collected. Often WDP will be conducted in the absence of a formal control group. Therefore, there is a need for other methods of evaluating the results. This could be various forms of benchmarking or other methodologies that allow for an interpretation of the effects of the program.

*Systematic presentation of on*-*going results that are relevant to employers*. There is a real need to connect better with employers to present results and underscore the value of scientific research. Likewise, scientists need to appreciate the perspective of employers in developing research programs.

## Conclusions

Evaluating the outcome of any preventive intervention program is integral for program development and the future choice of initiatives. We found that there are differences in the way the business community approaches outcome evaluation as opposed to how scientists approach this question. By integrating the two perspectives, outcome evaluation could be significantly improved. This is vital since the development and implementation of WDP programs depend on being able to evaluate their strengths and weaknesses. A clear step forward would be for these two communities to agree upon a basic set of outcome measures which would facilitate both perspectives and a multilevel evaluation. In addition, seeking the input of other stakeholder groups would further illuminate key player’s perspectives and priorities. In the end, all parties have much to gain by coordinating and integrating outcome evaluation.
